# Relationship Between Alcohol Consumption and Risky Sexual Behaviors Among Adolescents and Young Adults: A Meta-Analysis

**DOI:** 10.3389/ijph.2023.1605669

**Published:** 2023-04-19

**Authors:** Hyang-Soon Cho, Youngran Yang

**Affiliations:** ^1^ Department of Nursing, Graduate School, Jeonbuk National University, Jeonju, Republic of Korea; ^2^ College of Nursing, Research Institute of Nursing Science, Jeonbuk National University, Jeonju, Republic of Korea

**Keywords:** meta-analysis, risky sexual behavior, alcohol consumption, adolescent, young adult

## Abstract

**Objectives:** Adolescents exposed to alcohol have increased risky sexual behaviors (RSBs); however, the association between alcohol consumption and RSBs has to be systematically and quantitatively reviewed. We conducted a meta-analysis of the literature to systematically and quantitatively review the association between alcohol consumption and RSBs in adolescents and young adults.

**Methods:** We searched for qualified articles published from 2000 to 2020 and calculated pooled odds ratios (ORs) using the random-effect model. We also conducted meta-regression and sensitivity analyses to identify potential heterogeneity moderators.

**Results:** The meta-analysis of 50 studies involving 465,595 adolescents and young adults indicated that alcohol consumption was significantly associated with early sexual initiation (OR = 1.958, 95% confidence interval (CI) = 1.635–2.346), inconsistent condom use (OR = 1.228, 95% CI = 1.114–1.354), and having multiple sexual partners (OR = 1.722, 95% CI = 1.525–1.945).

**Conclusion:** Alcohol consumption is strongly associated with RSBs, including early sexual initiation, inconsistent condom use, and multiple sexual partners among adolescents and young adults. To prevent the adverse consequences of alcohol consumption, drinking prevention programs should be initiated at an early age and supported by homes, schools, and communities.

## Introduction

Alcohol consumption in adolescence can affect hormone secretion and the brain, disrupting secondary sex characteristics and damaging brain cells (1). Alcohol consumption at an early age causes disability and accounts for 13.5% of deaths among those aged 20 to 39, causing social and economic losses across our society along with health issues (2). According to the World Health Organization (WHO), 26.5% of all adolescents aged 15–19 years of age worldwide are current drinkers, which is approximately 155 million adolescents. The highest prevalence rates of current drinking are in the WHO European Region (43.8%), followed by the Region of the Americas (38.2%) and the Western Pacific Region (37.9%). While heavy episodic drinking (HED) is less common among adolescents than in the total population, it peaks at the age of 20–24 years, with prevalence rates among drinkers in this age group higher than in the total population, except in the Eastern Mediterranean Region. Among current drinkers aged 15–24 years, prevalence of HED is high among men, with some studies reporting rates as high as 54.2% (3).

Alcohol consumption during adolescence is one of the factors that increase risky sexual behaviors (RSBs) (4, 5). Adolescents with substance abuse issues, specifically alcohol abuse, had approximately twice as many sexual partners and were 70% more likely to be diagnosed with sexually transmitted diseases (STDs) than adolescents without substance abuse issues (6). According to the literature on the relationship between alcohol consumption and early sexual experience in middle school students: Yu et al (7) reported a 2.35-fold increase in early sexual initiation when drinking alcohol, Hong et al (8) reported a 1.43-fold increase, Ayhan et al (9) reported a 2.13-fold increase, and Bersamin et al. (10) reported a 7.4-fold increase.

Depending on the study, the age of first sexual experience among adolescents varies, with Korean adolescents having their first sexual experience at 13.2 years old on average (11), specifically 12.1 years for boys and 13.9 years for girls. Ayhan et al (9) indicated that the first sexual experience occurred before the age of 14 in most studies. In particular, 13.4% of female adolescents had their first sexual experience after drinking alcohol (5). This early sexual debut increases the risk of unwanted pregnancy and STDs (12). Adolescents not using a condom during sexual experiences was reported at 34.4% in a study by Wilson, Asbridge et al (13), 46.9% in a study by Rios-Zertuche et al (14), and 41.1% in a study by Mlunde et al (15), thereby demonstrating that the rate of condom use among adolescents is very low. Alcohol consumption in adolescents is one of the factors for increasing inconsistent condom use, and Sanchez et al (16) reported that 57.1% of adolescents with drinking alcohol experience did not use condoms during sexual intercourse. The most used definition of binge drinking in research is based on the National Institute on Alcohol Abuse and Alcoholism (NIAAA)’s definition of a pattern of drinking that brings a person’s blood alcohol concentration (BAC) to 0.08 g/dL or higher, which is typically reached after consuming 4 drinks for women or 5 drinks for men within 2 h (17). This definition is consistent with that used by Wilson et al (13) who found that binge drinking, as defined by the NIAAA, increased inconsistent condom use by 1.1 times among adolescents, and Sanchez et al (16) reported this to be 1.27 times. Inconsistent condom use in adolescents can increase the prevalence of STDs and lead to teenage pregnancies (18).

Alcohol consumption in adolescents increases the probability of having more than three sexual partners by 3.37 times (19). Particularly, compared to female adolescents, male adolescents have a 2.9-fold increase in the likelihood of having multiple sexual partners (13). Moreover, the number of sexual partners increased by 2.41 times in current binge drinkers compared to current light drinkers. Drinking alcohol during adolescence increases the likelihood of regretting having sexual intercourse (19), and if they continue to abuse alcohol after adolescence, these sexual behaviors will likely continue into adulthood (20).

Therefore, alcohol consumption during sexual intercourse reduces sexual inhibition and increases RSBs by impacting adolescents’ judgment. However, studies analyzing the relationship between alcohol consumption and RSBs among adolescents have measured alcohol consumption in various ways according to fragmentary questions, amount of alcohol consumption (glass) or frequency of alcohol consumption, and time point (past alcohol consumption and current alcohol consumption). As the results differ by sex, it is difficult to synthesize and summarize the factors affecting alcohol consumption. Additionally, no meta-analysis has integrated each sex behavior by separating early sexual debut, inconsistent condom use, and multiple sexual partners for RSBs, which has a higher risk of STDs and teenage pregnancies—rather than integrating alcohol consumption and sexual experience. Thus, this study conducted a meta-analysis to identify the integrated effect of alcohol consumption in adolescents and young adults on the three types of RSBs and to determine whether the effect differs according to sex (male and female). Additionally, this study focused on analyzing the relationship between early sexual initiation, inconsistent condom use, and multiple sexual partners among RSBs due to alcohol consumption, and aimed to provide the derived results as fundamental data for health education and program development for safe sexual behaviors and prevention of alcohol consumption among adolescents and young adults.

## Methods

### Design

We conducted a systematic review and meta-analysis, following the protocol reported in accordance with the Preferred Reporting Items for Systematic Review and Meta-Analysis Protocols (PRISMA-P) Statement (21). The study protocol was registered with the PROSPERO database under the registration number CRD42022301637.

### Search Strategy

Eight electronic bibliographic databases were used: Cumulative Index to Nursing and Allied Health Literature (CINAHL), Cochrane Library, EBSCO, PubMed, PsycINFO, Web of Science, Database Periodical Information Academic (DBpia, Korea), and Research Information Sharing Service (RISS, Korea). These were chosen since they comprehensively covered relevant literature in our field of study. Additionally, CINAHL, Cochrane Library, and PsycINFO are well-known and widely used databases in healthcare and psychology research, respectively. Korean databases, DBpia and RISS, allowed us to retrieve relevant studies published in the Korean language.

**FIGURE 1:**
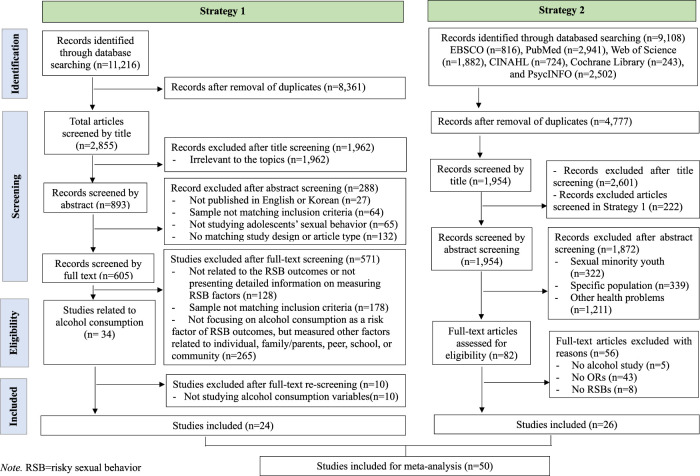
Flow chart of the selection process of studies included in the meta-analysis (Worldwide, 2001–2021).

We conducted a comprehensive literature review using two search strategies. The first strategy was to select studies that investigated the relationship between alcohol consumption and RSBs and included alcohol consumption as an independent variable affecting RSBs from large systematic reviews of RSB-related factors in individuals, peers, schools, and communities. We used the general keywords “factor(s) AND sexual behavior” or “risky sexual behavior” and “adolescent(s) or young adult(s)” through the title and abstract fields to identify relevant articles published between January 2000 and July 2020. The process of literature search, screening, and selection is presented in a separate research article (Ahn and Yang, 2022) for more detailed information. In the systematic review (Ahn and Yang, 2022), we included studies that examined the relationship between self-esteem and RSBs. However, for this meta-analysis, we selected studies that investigated the association between alcohol consumption and RSBs. This distinction is highlighted in the study inclusion criteria and is further explained in Figure 1.

The second search strategy used specific keywords to identify relevant articles. For example, in PubMed, we searched for articles using the following search terms: “Young Adult” [Mesh] OR “young adult*” OR “Adolescent” [Mesh] OR “adolescent*” OR “teen*” OR “youth*”), AND condom use (“Unsafe Sex” [Mesh] OR “unsafe sex” OR “unprotected sex” OR “high-risk sex” OR “unprotected intercourse” OR “condomless sex” OR “condom use” OR “condom compliance”) OR early sexual initiation (“Coitus” [Mesh] OR “Coitus” OR “First intercourse” OR “Sexual intercourse” OR “early sexual debut” OR “early sex*” OR “early sexual initiation”) OR multiple sexual partner (“Sexual Partners” [Mesh] OR “sexual partner*” OR “sex partner*” OR “multiple sexual partner*” OR “multiple sex partner*”).

### Inclusion/Exclusion Criteria

Full-text versions of the retrieved articles were screened based on the pre-specified inclusion and exclusion criteria. The inclusion criteria were as follows: 1) target population aged 15–24; 2) investigating the association between alcohol consumption and RSBs; and 3) defined early sexual debut as initiation before the age of 14 years. The exclusion criteria were: 1) if the target populations were sexual minority youth (e.g., gay, bisexual, lesbian, homosexual, men who have sex with men, or transgender); specific population (homeless, juvenile, or military); 2) focusing on HIV (human immunodeficiency virus), STI (Sexually transmitted infections), or other mental health problems; 3) did not report the odds ratio of early sexual debut, inconsistent condom use, multiple sexual partners separately; 4) did not report direct alcohol consumption association; and 5) insufficient information to calculate effect size or insufficient effect size to convert to odd ratio. We excluded studies that focused on sexual minority youth because our primary focus was on the general adolescent population. While we acknowledge the importance of understanding the relationship between alcohol consumption and risky sexual behavior among sexual minority youth, we considered that the sexual behavior of sexual minority individuals might differ from that of heterosexual individuals.

Finally, we identified 50 articles through the two search strategies, with 24 and 26 articles selected through the first and second strategies, respectively. This comprehensive approach enabled us to identify all relevant studies published between January 2000 and July 2020 that investigated the relationship between alcohol consumption and RSB.

### Data Extraction

Two independent researchers (CS and TK) conducted data extraction and study quality assessment, with a third researcher (YY) providing oversight to ensure accuracy and consistency. Any discrepancies between the two researchers were resolved through discussion and consensus. A standardized form was used to extract the following information: first author, year of publication, study country, total sample size, participant characteristics (e.g., mean age, gender distribution), measurements of alcohol consumption, and RSBs. OR and 95% CI for each type of RSB were abstracted by three researchers using a structured coding sheet. To reduce potential confounding variables and observational bias, adjusted estimates that controlled for demographic, individual, peer, school, and community variables were extracted if available. If multiple models were presented, we selected estimates from the last model that controlled for the most variables. This meta-analysis adhered to the Meta-Analysis of Observational Studies in Epidemiology Checklist, and the study selection flowchart was adapted from the Preferred Reporting Items for Systematic Reviews and Meta-Analyses statement (22, 23).

### Quality Assessment

The methodological quality of the primary studies in this meta-analysis was assessed using the Newcastle-Ottawa Scale (NOS) adapted for cross-sectional studies (24). The assessment tool consisted of seven items: selection (representativeness of the sample, sample size, non-respondents, ascertainment of the exposure (risk factor), comparability, and outcome (assessment of the outcome and statistical test). For each item criterion, a maximum of five points for selection, two points for comparability, and three points for outcome could be given and then summed to a maximum of 10 points. Quality assessment results were evaluated as “good” if the NOS score was 7 points or higher, “fair” if the score was 5 points or higher or less than 7 points, and “poor” if the score was less than 5 points. Each of the two researchers independently conducted the quality assessment of the studies included in the analysis, and the final assessment scores were obtained after discussing a few discrepancy items.

### Data Analysis

The data analysis was conducted using Comprehensive Meta-Analysis (CMA) Version 3 software (Biostat, Englewood, NJ, United States). Odds ratios and corresponding 95% confidence intervals were used to estimate the association between alcohol consumption and RSBs (early sexual initiation, inconsistent condom use, and multiple sexual partners). The extent of heterogeneity among the primary studies was assessed using Cochrane’s Q test (reported with x^2^ and *p* values) and Higgins I^2^ value (25). An I^2^ value of up to 25% indicated low heterogeneity, 50% indicated moderate heterogeneity, and ≥75% indicated high heterogeneity (25). Publication bias was evaluated using a funnel plot, trim-fill analysis, and Egger’s regression test. Sensitivity analysis was conducted by removing each study and examining the effect size (26). The random effects model was used for the meta-analysis, and a meta-regression analysis was performed with sex as a control variable. The level of significance for all analyses in this study was determined using a two-tailed *p* value of <0.05 with 95% confidence intervals.

## Results

### Characteristics of Primary Studies

Fifty studies were included in the meta-analysis based on the selection criteria of this study and the Newcastle-Ottawa Scale (NOS) assessment (Table 1). These studies were published between 2002 and 2021, and among the included studies, most were published in 2009, 2013 (*n* = 6 studies), followed by 2010 (*n* = 5 studies), 2017 (*n* = 4 studies), 2018 (*n* = 4 studies), and 2021 (*n* = 4 studies). Most published studies were written in English (*n* = 47 studies), and three were written in Korean; among the 50 articles, most studies were conducted in the United States (*n* = 23 studies), and seven were conducted in Europe (Croatia, Finland, Ireland, and the United Kingdom) and Asia (South Korea, China, Hong Kong, and Japan).

**TABLE 1:** Characteristics of the included studies on the relationship between alcohol consumption and RSB (Worldwide, 2001–2021).

# of Reference	Author (year)	Country	Sample (N)	Study design/Data source	Mean age (range)	% of gender (M/F)	Measurement of alcohol consumption		Measurement of RSBs		Type of RSB
(27)	Mo et al. (2007)	Canada	N = 44,430	A cross-sectional study	Mean age = Not presented (12–19 years)	M (47.7%) F (52.3%)	Ever drink alcohol (yes/no)		2. Inconsistent condom use: - Have not always used a condom during sexual intercourse in the past 12 months 3. Multiple sexual partners: Two or more sexual partners in the past 12 months		2, 3
(28)	Lepusic et al. (2013)	Croatia	N = 715	A longitudinal study	Mean age = 17.9 (15–21 years)	F (100.0%)	Alcohol use -“How many alcoholic drinks do you usually have at the time?”		2. Inconsistent condom use		2, 3
									3. Multiple sexual partners during the past 60 days (yes/no)		
(14)	Rios-Zertuche et al. (2017)	Croatia	N = 919	A cross-sectional study	Mean age = 14.7 (12–19 years)	M (41.5%)	Excessive alcohol consumption		2. Used a condom when you last had sex		2
						F (58.5%)					
(29)	Scott-Sheldon et al. (2010)	United States	N = 221	A cross-sectional study	Mean age = 19 (18–25 years)	F (67.0%)	Alcohol consumption		2. Condom use during vaginal or anal sex		2
						M(33.0%)					
(30)	Mola et al. (2017)	Brazil	N = 1,275	A cross-sectional study	Mean age = Not presented (12–24 years)	Not presented	Alcohol consumption during lifetime -non-user ∼ never drank alcohol during lifetime		3. Number of sexual partners in lifetime -“with how many different people have you had sexual intercourse during your lifetime?”		3
(13)	Wilson et al. (2010)	Canada	N = 2,297	A cross-sectional study	Mean age = 16.6 (9–12 Grade)	F (51.0%) M(49.0%)	Alcohol use -having had 5 or more drinks on 2 or more occasions in the past month		2. Condom non-use: Not using a condom during last vaginal intercourse		2
(15)	Mlunde et al. (2012)	Tanzania	N = 2,217	A cross-sectional study	Mean age = Not presented (15–24 years)	F (39.8%) M (60.2%)	Alcohol use in the past 1 year (yes/no)		2. Condom use during last sexual intercourse		2
(31)	Kang et al. (2013)	Jamaica	N = 330	A longitudinal study	Mean age = 14.7 (13–17 years)	F (100.0%)	Alcohol use -“In the past 3 months, how often did you drink beer, wine or liquor?”		2. Condom use during last sexual intercourse (no vs. yes) -“did you use a condom?”		2
(32)	Kaltiala et al. (2015)	Finland	N = 2,070	Prospective cohort study	Mean age = 17.6 (All ninth graders)	F (56.6%) M (43.4%)	Frequent alcohol use -“How often do you drink alcoholic beverages?”		3. Intercourse with five or more partners -“With how many partners have you had sexual intercourse?” (one/two/three/four/five or more)		3
(33)	Ramisetty et al. (2004)	United States	N = 2,657	A three-stage cluster sample design	Mean age = Not presented (9–12 grade)	Not presented	Alcohol use and drinking pattern		3. Multiple partners (lifetime and recent) - Number of people they had sex with in their lifetime and during the past 3 months (recent)		3
(34)	Kugbey et al. (2018)	Ghana	N = 1,648	A secondary analysis of data from GSHS	Mean age = Not presented (11–18 years)	F (47.5%) M (52.5%)	Alcohol use		2. Condom use during last sexual intercourse -The last time you had sexual intercourse, did you or your partner use a condom or rubber? 3. Multiple sexual partners - during your life, how many people have you had sexual intercourse with?		2, 3
(35)	Ronis et al. (2011)	Canada	N = 1,100	A cross-sectional study	Mean age = 14.8 (grades 9–11; 13–16 years)	F (64.7%) M (35.3%)	Lifetime use of alcohol		1. Age of first sexual intercourse (13–16 years)		1
(9)	Ayhan et al. (2015)	South America	N = 1,603	A cross-sectional study	Mean age = 15 (13–18 years)	F (48%) M (52%)	Alcohol		1. Early first SI (sexual intercourse) - SI at the age of 15 years or before was defined as premature		1
(18)	Menna et al. (2014)	Ethiopia	N = 1,948	A cross-sectional study	Mean age = Not presented (15–24 years)	F (60.7%) M (39.3%)	Drinking alcohol (yes/no)		3. Number of sexual partners -Number of sexual partners in previous year -Two or more		3
(36)	Peltzer et al. (2016)	Oceania (Fiji, Kiribati, Samoa, and Vanuatu)	N = 6,792	A cross-sectional study	Mean age = Not presented (13–16 years)	F (50.3%) M (49.7%)	Alcohol use -“During the past 30 days, how many days did you have at least one drink containing alcohol?” - 1 = 0 days to 7 = All 30 days		1. Age of sexual debut - “How old were you when you had sexual intercourse for the first time?” - Early Sexual Debut (<14 Years) 2. Condom use -“The last time you had sexual intercourse, did you or your partner use a condom?” - Condom during last Sexual intercourse 3. Number of sexual partners - “During your life, how many people have you had sexual intercourse with?” - Two or more sexual partners		1, 2, 3
(37)	Kogan et al. (2010)	United States	N = 292	A cross-sectional study	Mean age = Not presented (18–21 years)	F (59.9%) M (40.1%)	Binge drinking -Four or more drinks at one sitting		2. Unprotected intercourse -condom use at sexual intercourse during the past 3 months		2
(8)	Hong et al. (2018)	Republic of Korea	N = 68,043	A secondary analysis of data from KYRBWS	Mean age = 13.56 (14–16 years)	Not presented	Alcohol consumption		1. Coital experience (14–16)		1
(38)	Connor et al. (2013)	New Zealand	N = 2,921	A cross sectional web-based survey	Mean age = Not presented (17–25 years)	F (61.0%) M (39.0%)	Alcohol consumption - Questionnaire (Bohn et al., 1995) were used to assess alcohol consumption in the previous 4-week period on a scale of 0–12 (AUDIT-C) - 1. How often do you have a drink containing alcohol? −2. How many drinks do you have on a typical day when you are drinking? - 3. How often have you had 6 or more drinks on one occasion?		2. Condom use at last sexual intercourse Did you use a condom the last time you had sex? (yes, no)		2
(39)	Seidu et al. (2021)	Namibia	N = 4,531	A secondary analysis of data from GSHS	Mean age = Not presented (grade 6–12)	Not presented	Alcohol use -During the past 30 days, on how many days did you have at least one drink containing alcohol?		3. Multiple sexual partners - During your life, how many people have you had sexual intercourse with?		3
(40)	Yarber et al. (2002)	United States	N = 1,130	A secondary analysis of data from YRBS	Mean age = M (15.5 years) F (15.4 years) (14–18 or older)	F (50.4%) M (49.6%)	Binge drank past 30 days		3. Multiple sexual partners during one’s lifetime - having two or more sexual partners during one’s lifetime		3
(41)	Benotsch et al. (2013)	United States	N = 763	A cross-sectional study	Mean age = 18.9 years (18–25 years)	Not presented	Alcohol use - The frequency of use of alcohol in the previous 3 months		2. Total number of unprotected sex (no condom used) - Vaginal or anal sex acts in the past 3 months 3. Number of sexual partners during one’s lifetime		2, 3
(16)	Sanchez et al. (2013)	Brazil	N = 17,371	A cross sectional study	Mean age = Not presented (13–18 years)	Not presented	Alcohol use		2. Sexual intercourse without condom use -Sexual intercourse without condom use during the past month (30 days)		2
(42)	Lee et al. (2010)	United States	N = 1,073	National longitudinal study of Add Health	Mean age = Not presented (18–27 years)	Not presented	Binge drinking - “Over the past 12 months, how many days did you drink alcohol (five or more drinks in a row?)”		2. Not using condom during recent sexual intercourse - “The most recent time you had vaginal intercourse—did you/your partner use a condom?’’ 3. Four or more sexual partners during one’s lifetime - “How many partners have you ever had vaginal intercourse with? Even if only once.”		2, 3
(43)	Agu et al. (2018)	Jamaica	N = 3,365	A secondary analysis of data from NSS	Mean age = = Not presented (grade 8, 10, 11, 12)	Not presented	1. Lifetime alcohol consumption 2. Alcohol consumption: past 12 months 3. Alcohol consumption: past 30 days		2. Non-condom use -non-condom use during sex		2
(44)	Bartholomew et al. (2021)	London	N = 509	A cross sectional study	Mean age = 17 M (18.1 years) F (17.8 years) (16–24 years)	F (53.0%) M (47.0%)	At least one episode of being drunk in the past month		3. ≥2 sexual partners in the past year		3
(45)	Brown et al. (2007)	United States	N = 547	A cross-sectional study	Mean age = Not presented (18 or older)	Not Presented	Alcohol use - Consumed alcohol: number of drinks consumed		2. Unprotected vaginal sex during the most recent sexual encounter -Their most recent sexual experience, including items assessing which sexual behaviors occurred (i.e., oral, vaginal, and anal sex) -Respondents also indicated if a condom was used		2
(46)	Kiene et al. (2009)	United States	N = 116	A longitudinal cohort daily diary study	Mean age = 19.15	F (57.8%) M (42.2%)	Daily alcohol consumption (number of drinks) - The number of drinks 30 consumed “last night.”		2. Unprotected sex - Unavailability of condoms		2
(47)	Adhikari (2010)	Nepal	N = 1,137	A cross sectional study	Mean age = Not presented (Grades 11 and 12; 15–19 or 20 and above	F (49.6%) M (50.4%)	Alcohol consumption		2. Condom use - Condom use during first sexual intercourse		2
(10)	Bersamin et al. (2006)	United States	N = 1,105	A longitudinal study	Mean age = 14.1 (12–16 years)	F (48.2%) M (51.8%)	Heavy drinking - How often, during the past 30 days, had the respondents had five or more alcoholic drinks within a 2-h period		1. Sexual activity (sexual intercourse) -Vaginal sex (“Have you ever had sexual intercourse?”) -Yes/no		1
(48)	Nkansah-Amankra et al. (2011)	United States	N = 1,474	A cross sectional data from the YRBS	Mean age = Not presented (grade 9–12)	F (49.1%) M (50.9%)	1. Alcohol use - Non-drinkers (no alcohol in the past 30-day period), current light drinkers (1 drink in the past 30 days but 5 drinks in a row), and current binge drinkers (5 or more drinks in a row)		1. Age at first sexual intercourse - Young adolescents (11–14 years) 3. Number of current sexual partners - Number of sexual partners in the past 3 months - >2 Sexual partners		1, 3
(56)	Page and Hall (2009)	Africa	N = 22,949	A secondary analysis of data from GSHS	Mean age = Not presented (13–15 years)	Not presented	Alcohol use -“During the past 30 days, on how many days did you have at least 1 drink containing alcohol?		1. “How old were you when you had sexual intercourse for the first time?” (had sex before age 13)		1
(7)	Yu et al. (2014)	Korea	N = 37,297	A cross sectional study	Mean age = Not presented (12–15 years)	F (48.3%) M (51.7%)	Present alcohol consumption -”In the past 30 days, on how many days did you drink at least one drink?”		1. Sexual debut - Sexual intercourse with same or opposite sex		1
(50)	Gwon et al. (2015)	Korea	N = 74,186	A secondary analysis of data from Korea Youth Risk Behavior Web-based Survey	Mean age = Not presented (12–15 years)	F (47.9%) M (52.1%)	Ever drinking		1. Sexual debut - Sexual intercourse with same or opposite sex		1
(51)	Brouwer et al. (2019)	United States	N = 241	A cross-sectional study	Mean age = 18.9 (18–22 years)	F (69.7%) M (30.3%)	Alcohol use		3. Number of sexual partners - Sexual contact as vaginal intercourse, oral sex, or anal sex and defines a sexual partner as an individual with whom one engages in one or more of these acts		3
(52)	Choi et al. (2016)	HongKong	N = 666	A cross sectional study	Mean age = 20.03	F (54.1%) M (44.4%) [*did not answer (1.5%)]	Drinking habit		2. Condom use - Consistent vs. inconsistent - Condom use during the last sexual intercourse		2
(53)	Lewis et al. (2012)	United States	N = 1,468	A cross-sectional study	Mean age = 19.9 (18–25 years)	F (56.4%) M (43.6%)	Drinking during hookup - Number of drinks they had directly prior to or during the most recent hookup		2. Condom use - “During this hookup, did you or your partner use a condom?”		2
(54)	Kuortti et al. (2009)	Finland	N = 247	A cross sectional study	Mean age = 17.27 (15–18 years)	F (100.0%)	Binge drinking		3. Multiple sexual partners		3
(55)	Lavikainen et al. (2009)	Finland	N = 100,790	A secondary analysis of data from SHPS	Grade 8 (50,569) -Mean age = 14.8 Grade 9 (48,132) Mean age = 15.8	Grade 8 -F (49.6%) -M (50.4%) Grade 9 -F (48.9%) -M (51.1%)	Drinking style 1) Frequency of drinking alcohol: “How often do you use alcohol?” 2) Drunkenness related drinking: frequency of drunkenness: “How often do you use alcohol until you are really drunk?”		3. Three or more sexual partners -“How many different partners have you had sexual intercourse with?”		3
(5)	Young et al. (2018)	Ireland	N = 4,494	A cross-sectional study	Mean age = Not presented (15–18 years)	F (46.2%) M (53.8%)	Alcohol involvement		1. Sexual initiation (very early sexual initiation (<14 years) - “Have you ever had sexual intercourse?” -“How old were you when you had sexual intercourse for the first time?”		1
(4)	Pengpid et al. (2021)	Africa (Mozambique)	N = 1,918	A secondary analysis of data from GSHS	Mean age = Not presented (11–18 years)	Not presented	Current alcohol use - “During the past 30 days, on how many days did you have at least one drink containing alcohol?”		2. Condom use - “The last time you had sexual intercourse, did you or your partner use a condom?” 3. Number of sexual partners - “During your life, how many people have you had sexual intercourse with?”		2, 3
(49)	Patrick et al. (2009)	United States	N = 218	A cross-sectional study	Mean age = 18.85 (18.12–20.74 years)	Not presented	Alcohol use, - Number of standard drinks consumed the day prior		2. Condom use with penetrative sex - Condom use was coded only for days with penetrative sex as 0 = no and 1 = yes		2
(57)	Woodrome et al. (2006)	United States	N = 117	A cross-sectional study	Mean age = 16.2 (14–17 years)	F (100.0%)	Alcohol use concordance		2. Condom non-use		2
(19)	Agius et al. (2013)	Australia	N = 450	A secondary analysis of data from IYDS	Mean age = 17.0 (Grade 5, 7 and 9)	F (56.0%) M (44.0%)	Drinking 1) Binge drinking: -”Think back over the past 2 weeks, how many times have you had five or more drinks in a row?” 2) Compulsive drinking -“When drinking alcohol over the past year, have you ever found that you were not able to stop drinking once you had started?”		2. Condom non-use at last sexual encounter - “The last time you had sex, did you or your partner use a condom or another latex barrier?” 3. Three or more sexual partners in the past year - “In the past year, how many males and/or females have you had sex with?”		2, 3
(58)	Guo et al. (2017)	China	N = 22,288	A secondary analysis of data from YARHC	Mean age = 19.0 (15–24 years)	F (49.7%) M (50.3%)	Only alcohol use (OAU)			2. Condom non-use during first sexual experience 2. Condom non-use during most recent sexual encounter 3. Sexual activity with multiple partners	2, 3
(59)	Seth et al. (2011)	United States	N = 393	A cross-sectional study	Mean age = 17.9 (15–21 years)	F (100.0%)	Alcohol Use -“How many alcoholic drinks do you usually have at one time?”			2. Inconsistent condom use 3. Multiple sexual partners during the past 60 days	2, 3
(60)	Cavazos-Rehg et al. (2007)	United States	N = 601	A cross-sectional study	Mean age = 21.0 (18–25 years)	F (50.4%) M (49.6%)	Alcohol involvement			3. Number of Sexual Partners in lifetime >10 - “How many sexual partners have you had in your life?”	3
(61)	Thompson et al. (2005)	United States	N = 17,264	A secondary analysis of data from Department of Defense Survey of Health	Mean age = Not presented (18 ∼ >46 years)	F (14.0%) M (86.0%)	Frequency of alcohol intoxication - How often they got drunk or very high from drinking in the past 12 months			2. Frequency of condom use with casual and one-time sexual partners in the past 12 months 3. Number of sexual partners in the past 12 months	2, 3
(62)	Hurley et al. (2017)	Republic of the Congo	N = 1,396	A cross-sectional study	Mean age = 18 (15–24 years)	F (57.3%) M (42.7%)	Alcohol use			1. Sexual debut under age 15	1, 2, 3
										2. Unprotected sex	
										- Inconsistent or no condom use with one or more partners in the last 12 months	
										3. Multiple sexual partners	
										-Two or more sexual partners in the last 12 months	
(63)	Scroggins et al. (2021)	US Cross-sectional analysis: utilized data from the 2017 National Youth Risk Behavioral Survey (YRBS)	N = 3,732	A secondary analysis of data from Department of Defense Survey of YRBS	Mean age = Not presented (US high school students)	F (51.0%), M (49.0%)		Alcohol use pattern (past 30 days)		2. Condom utilization— “The last time you had sexual intercourse, did you or your partner use a condom?”	2
(64)	Izutsu et al. (2009)	Japan	N = 281	A cross-sectional study	Mean age = 16.88 (13–20 years)	M (100.0%)		AAIS (Adolescent Alcohol Involvement Scale)		3. Number of sexual partners (0 = less than 2 and 1 = more than 3	3

Notes: RSB, risky sexual behaviors; Type of RSB (1): Early sexual initiation (2), Inconsistent condom use (3), Multiple sexual partners.

The number of participants in 50 studies was 442,909, with sample sizes ranging from 116 to 100,790; whereas others used large-scale data from the School Health Promotion Study (SHPS) in Finland or the Youth Risk Behavior Surveillance (YRBS) developed by the Centers for Disease Control and Prevention (CDC) of the United States (55, 63). SHPS is a nationally representative sample of adolescents born between 1987 and 1989, with 374 municipalities out of 448 participating in Finnish municipalities. The YRBS is weighted by respondents to be nationally representative data from the 2017 National YRBS cross-sectional data developed by the US CDC for US high school students (*n* = 3,732).

The overall mean age of the participants was 17.24 years, and most studies were conducted with both males and females (*n* = 34 studies). However, some studies focused on a specific sex, such only females (*n* = 5 studies) and only males (*n* = 1 study).

Exploring the characteristics of the responses used in the measurement of alcohol consumption, the most common response was a “yes” or “no” for alcohol drinking (*n* = 28 studies), and the measurement methods of alcohol use were different for each study, such as frequency of alcohol consumption (*n* = 6 studies), amount of alcohol consumption (glass) (*n* = 4 studies), alcohol involvement (*n* = 2 studies), drinking pattern (*n* = 2 studies), and others (*n* = 6 studies). Some studies used the Alcohol Use Disorders Identification Test (AUDIT-C) to assess alcohol consumption in the previous 4-week period on a scale of 0–12 (AUDIT-C) (38), while another study used the Adolescent Alcohol Involvement Scale (AAIS) tool (64).

### Quality of the Included Studies

Based on the Newcastle-Ottawa Scale assessment, 31 articles were rated as “good” while the remaining studies were rated as “fair” in terms of methodological quality (Table 2).

**TABLE 2:** Quality of the included studies (Newcastle-Ottawa Scale) (Worldwide, 2001–2021).

# of Reference	Author(s) (year)	Selection				Comparability	Outcome		Quality Score
		Representativeness of the sample	Sample size	Non-respondents	Ascertainment of the exposure	Based on design and analysis	Assessment of the outcome	Statistical test	
(27)	Mo et al. (2007)	*	*		*	*	*	*	6
(28)	Lepusić et al. (2013)	*	*	*	*	*	*	*	7
(14)	Rios-Zertuche et al. (2017)	*	*		*	*	*	*	6
(29)	Scott-Sheldon et al. (2010)	*	*	*	*	*	*	*	7
(30)	Mola et al. (2017)	*	*		**	*	*	*	7
(13)	Wilson et al. (2010)	*	*	*	*	*	*	*	7
(15)	Mlunde et al. (2012)	*	*		**	*	*	*	7
(31)	Kang et al. (2013)	*	*		*	*	*	*	6
(32)	Kaltiala et al. (2015)	*	*	*	*	*	*	*	7
(33)	Ramisetty et al. (2004)	*	*	*	*	*	*	*	7
(34)	Kugbey et al. (2018)	**	*	*	**	*	*	*	9
(35)	Ronis et al. (2011)	*	*	*	*	*	*	*	7
(9)	Ayhan et al. (2015)	**	*			*	*	*	6
(18)	Menna et al. (2014)	*	*	*	*	*	*	*	7
(36)	Peltzer et al. (2016)	**	*	*	**	*	*	*	9
(37)	Kogan et al. (2010)		*		*	*	*	*	5
(8)	Hong et al. (2018)	*	*	*	*	*	*	*	7
(38)	Connor et al. (2013)	*	*		**	*	*	*	7
(39)	Seidu et al. (2021)	*	*	*	*	*	*	*	7
(40)	Yarber et al. (2002)	*	*	*	*	*	*	*	7
(41)	Benotsch et al. (2013)	*	*	*	*	*	*	*	7
(16)	Sanchez et al. (2013)	*	*	*	**	*	*	*	8
(42)	Lee et al. (2010)	*	*		*	*	*	*	6
(43)	Agu et al. (2018)	*	*			*	*	*	5
(44)	Bartholomew et al. (2021)	*	*		*	*	*	*	6
(45)	Brown et al. (2007)	*	*		*	*	*	*	6
(46)	Kiene et al. (2009)	*	*		*	*	*	*	6
(47)	Adhikari. (2010)	*	*		**	*	*	*	7
(19)	Bersamin et al. (2006)	*	*	*	*	*	*	*	7
(48)	Nkansah-Amankra et al. (2011)	*	*	*	*	*	*	*	7
(49)	Patrick et al. (2009)	*	*	*	*	*	*	*	7
(7)	Yu et al. (2014)	**	*	*	*	*	*	*	8
(50)	Gwon et al. (2015)	**	*	*	*	*	*	*	8
(51)	Brouwer et al. (2019)	*	*		*	*	*	*	6
(52)	Choi et al. (2016)	*			*	*	*	*	5
(53)	Lewis et al. (2012)	*	*		*	*	*	*	6
(54)	Kuortti et al. (2009)	*	*	*	*	*	*	*	7
(55)	Lavikainen et al. (2009)	*	*		*	*	*	*	6
(5)	Young et al. (2018)	*	*	*	*	*	*	*	7
(4)	Pengpid et al. (2021)	**	*	*	*	*	*	*	8
(56)	Page and Hall (2009)	**	*	*	*	**	*	*	9
(57)	Woodrome et al. (2006)	*	*		*	*	*	*	6
(19)	_Agius et al. (2013)_	**	*	*	*	*	*	*	8
(58)	_Guo et al. (2017)_	*	*	*	*	*	*	*	7
(59)	Seth et al. (2011)	*	*	*	*	*	*	*	7
(60)	Cavazos-Rehg et al. (2007)	*			*	*	*	*	5
(61)	Thompson et al. (2005)		*		*	*	*	*	5
(62)	Hurley et al. (2017)	*	*		*	*	*	*	6
(63)	Scroggins and Shacham (2021)	*	*		**	*	*	*	7
(64)	Izutsu et al. (2009)	*			**	*	*	*	6

### Results of Meta-Analysis

#### Early Sexual Initiation

**FIGURE 2:**
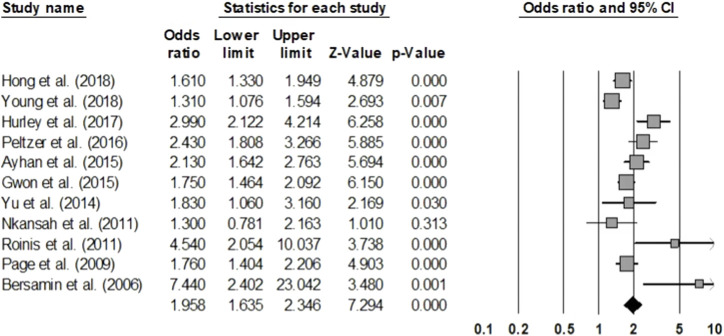
Forest plot (Early sexual initiation) (Worldwide, 2001–2021).

After analyzing 11 articles studying early sexual initiation in adolescents, the incidence of sexual experience at an early age was found to be 1.958 times higher in the group that consumed alcohol compared to the group that did not (OR = 1.958, 95% CI = 1.635–2.346) (Figure 2). The analysis was based on 220,439 adolescents across the studies. The homogeneity test resulted in a Q statistic of 38.278 (*p* < 0.001) and an I^2^ statistic of 73.88%, demonstrating relatively high heterogeneity. Hence, the effect size was analyzed using a random-effects model.

The publication bias test showed that some studies were omitted to the left of the mean effect size in the funnel plot, which seemed to be asymmetric, and Egger’s regression test showed a publication error (*t* = 2.272, *p* = 0.049). When the publication error is corrected using the trim-and-fill method, it is still statistically significant even if one more study is added (OR = 1.906, 95% CI = 1.581–2.298). Additionally, the sensitivity analysis showed that there was no substantial difference in the range of the mean effect size even after removing each individual article (1.854–2.056); and all were significant. To explain the heterogeneity of the effect size, a meta-regression analysis with sex as a moderating variable was conducted, indicating that it was statistically insignificant (Z = 0.600, *p* = 0.548) (Table 3).

**TABLE 3:** Results of meta-regression (Early sexual initiation) (Worldwide, 2001–2021).

Gender	Subgroup analysis	Meta-regression				
	OR (95% CI)	Covariate	B	SE	95% CI	Z (p)
		Intercept	0.480	0.145	(0.195, 0.764)	3.300 (<0.001)
Females	1.615 (1.214, 2.149)	Females	1.000			
Males	1.814 (1.412, 2.332)	Males	0.116	0.194	(−0.263, 0.496)	0.600 (0.548)

Notes: 6 articles included in the subgroup analysis.

#### Inconsistent Condom Use

**FIGURE 3:**
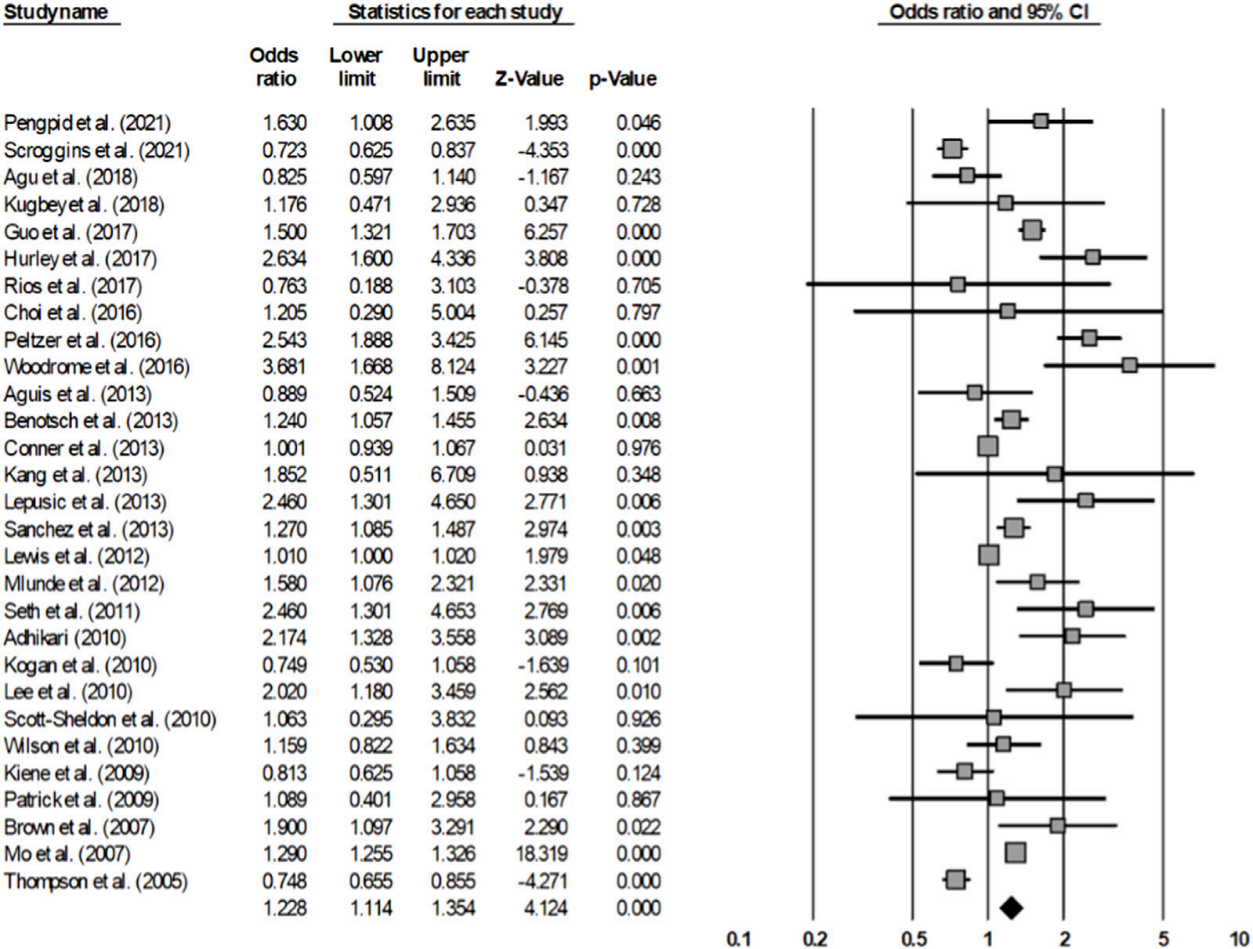
Forest plot (Inconsistent condom use) (Worldwide, 2001–2021).

After analyzing 29 articles that investigated inconsistent condom use among adolescents and young adults, the rate of condom non-use during sexual intercourse was found to be 1.228 times higher in the group that consumed alcohol compared to the group that did not consume alcohol (OR = 1.228, 95% CI = 1.114–1.354) (Figure 3). The analysis included 137,064 individuals across the studies. The homogeneity test resulted in a Q statistic of 474.933 (*p* < 0.001), and an I^2^ statistic of 94.10%, demonstrating high heterogeneity. Hence, the effect size was analyzed using a random-effects model.

The publication bias test showed that some studies were omitted to the left direction of the mean effect size in the funnel plot, which seemed to be asymmetric; however, it can be determined that there was no publication error according to Egger’s regression test (*t* = 1.732, *p* = 0.095). Additionally, the sensitivity analysis showed that there was no substantial difference in the range of the mean effect size, even after removing each article (1.184–1.283); and all were significant. To explain the heterogeneity of the effect size, a meta-regression analysis with sex as a moderating variable was conducted, which indicated that it was statistically insignificant (Z = −0.700, *p* = 0.4817) (Table 4).

**TABLE 4:** Results of meta-regression (Inconsistent condom use) (Worldwide, 2001–2021).

Gender	Subgroup analysis	Meta-regression				
	OR (95% CI)	Covariate	B	SE	95% CI	Z (p)
		Intercept	0.302	0.190	(-0.070, 0.675)	1.590 (0.112)
Females	1.353 (0.938, 1.950)	Females	1.000			
Males	1.134 (0.820, 1.567)	Males	−0.177	0.251	(-0.670, 0.316)	−0.700 (0.482)

Notes: 7 articles included in the subgroup analysis.

#### Multiple Sexual Partners

**FIGURE 4:**
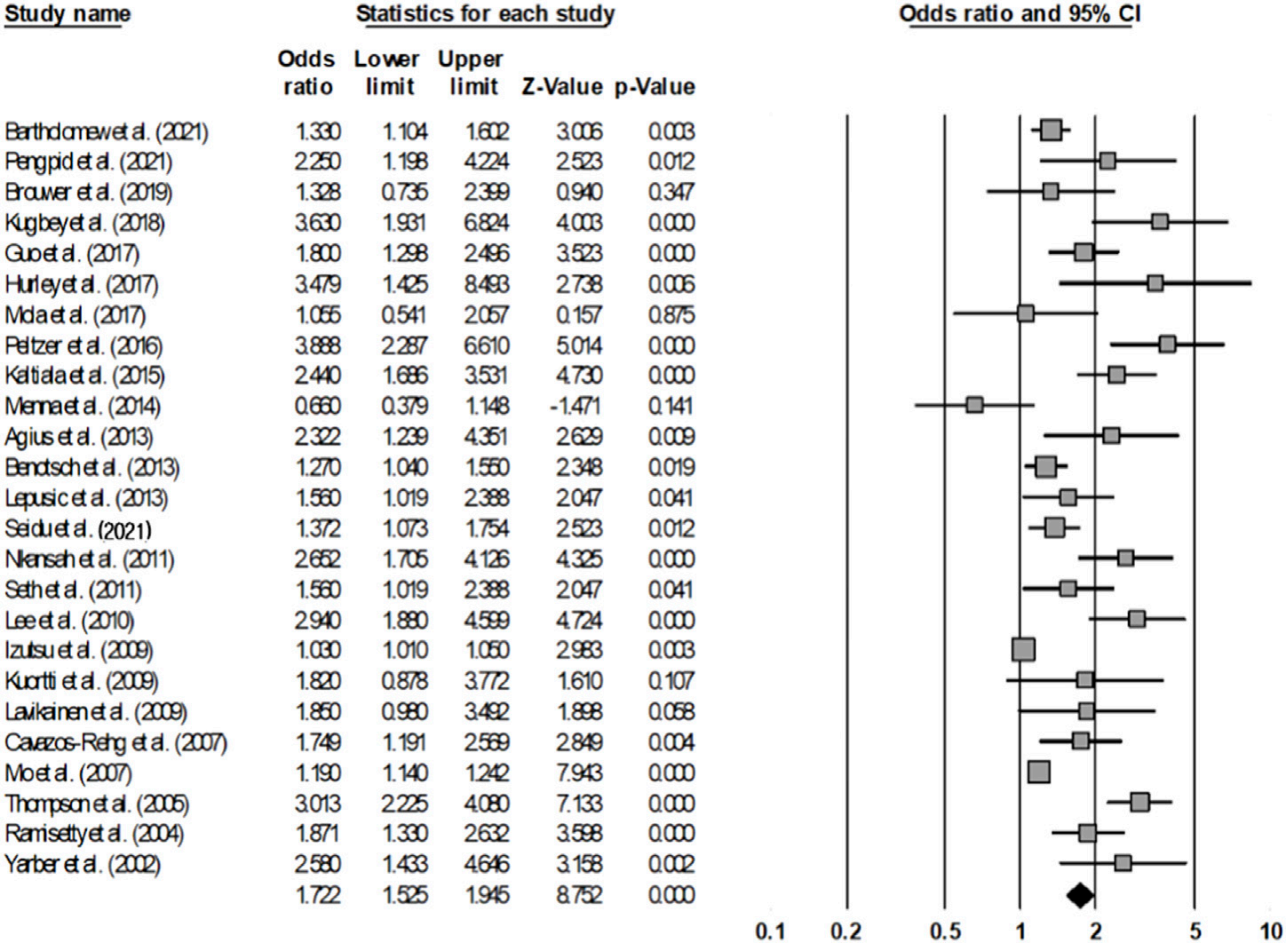
Forest plot (Multiple sexual partners) (Worldwide, 2001–2021).

Upon analyzing 25 articles, based on a total of 216,884 adolescents and young adults, that studied multiple sexual partners, the rate of having multiple sexual partners was 1.722 times higher in the group that consumed alcohol than in the group that did not (OR = 1.722, 95% CI = 1.525–1.945) (Figure 4). The homogeneity test resulted in a Q statistic of 253.151 (*p* < 0.001) and an I^2^ statistic of 90.52%, demonstrating high heterogeneity. Hence, the effect size was analyzed using a random-effects model.

The publication bias test showed that some studies were omitted to the left direction of the mean effect size in the funnel plot, which seemed to be asymmetric, and there was a publication error according to Egger’s regression test (*t* = 6.518, *p* < 0.001). When the publication error is corrected using the trim-and-fill method, it can be seen that it is still statistically significant even if nine more studies are added (OR = 1.367, 95% CI = 1.224–1.527). In addition, the sensitivity analysis showed that there was no substantial difference in the range of the mean effect size even after removing each article (1.653–1.838); and all were significant.

To explain the heterogeneity of the effect size, a meta-regression analysis with sex as a moderating variable was conducted, indicating that it was statistically insignificant (Z = −0.340, *p* = 0.732) (Table 5).

**TABLE 5:** Results of meta-regression (Having multiple sexual partners) (Worldwide, 2001–2021).

Gender	Subgroup analysis	Meta-regression				
	OR (95% CI)	Covariate	B	SE	95% CI	Z (p)
		Intercept	0.913	0.194	(0.532, 1.294)	4.700 (<0.001)
Females	2.590 (2.022, 3.317)	Females	1.000			
Males	2.262 (1.405, 3.640)	Males	0.094	0.275	(-0.633, 0.445)	−0.340 (0.732)

Notes: 6 articles included in the subgroup analysis.

## Discussion

This meta-analysis included 50 independent studies that investigated the relationship between alcohol consumption and RSBs in adolescents and young adults. The RSBs considered in this study were early sexual initiation, inconsistent condom use, and multiple sexual partners. The odds ratio (OR) was used as the effect size measure, with OR = 1.958 for early sexual initiation, OR = 1.228 for inconsistent condom use, and OR = 1.722 for multiple sexual partners among adolescents and young adults who consumed alcohol, compared to those who did not. No sex-based differences were observed. These findings are consistent with the Alcohol Myopia Theory (65), which posits that alcohol consumption can impair cognitive processing and narrow individuals’ focus of attention rather than long-term goals or consequences, leading to a myopic view of the immediate environment and an over-reliance on cues that are immediately present, which may make individuals less likely to consider the potential consequences of their actions and more likely to engage in risky behaviors (66). By understanding the mechanisms underlying the association between alcohol consumption and RSBs, we can develop more effective prevention and intervention strategies to reduce the negative health outcomes associated with these behaviors.

The age of early sexual initiation among adolescents can vary within and between countries and cultures, but the mean age of sexual intercourse among sexually-experienced adolescents is typically between the ages of 14 and 15 (9, 10, 35). Some studies define early sexual experience as occurring at 14 years of age or younger (5, 36), but it is notable that the trend of sexual initiation age is rising in many countries. For example, a study conducted in the United States found that the proportion of adolescents who have had sexual intercourse before the age of 13 has declined over time from 10.2% in 1991% to 3.4% in 2017 (67). Similarly, a recent study on sub-Saharan Africa found that the age at first sexual intercourse among birth cohorts entering adulthood between 1985 and 2020 had increased over time, with the median age at first sex rising from 17.1 to 18.7 years for men and from 17.6 to 19.1 years for women (68).

The odds ratio of alcohol consumption and early sexual initiation was the highest at 1.958. This phenomenon of early alcohol consumption and risky behaviors seems to be particularly prominent in the United States, where the minimum legal drinking age (MLDA) is 21 years old (69). Despite this, a significant number of high school students under the age of 21 still report drinking alcohol, with one study finding that 29% of high school students reported consuming alcohol (67). Mo et al (27) also reported that 43.4% of adolescents between the ages of 14 and 16 had consumed alcohol and were 1.13 times more likely to engage in early sexual initiation. Early alcohol consumption during adolescence can lead to other risky behaviors, such as smoking, substance abuse, and RSBs (17, 69). Thus, implementing policies and strategies to increase the age at which alcohol is initiated, and measures like designating the MLDA and requiring ID for alcohol purchases, could be effective in reducing alcohol consumption among young people. However, it is notable that further research is needed to better understand the complex relationship between alcohol consumption and risky behaviors among adolescents and young adults.

Of the 50 studies analyzed in the meta-analysis, 29 studies examined the relationship between inconsistent condom use and alcohol consumption among adolescents. The results showed that adolescents and young adults who drank were 1.228 times more likely to engage in inconsistent condom use during sexual intercourse that those who did not.

These findings are consistent with the Inhibition Conflict Theory, which suggests that alcohol consumption can lead to a conflict between an individual’s desires and their inhibitions, resulting in a failure to inhibit inappropriate or risky behaviors (70). Dermen and Cooper (70) provides further support for this theory, showing that the quantity of alcohol consumed was negatively associated with condom use only among high-conflict individuals. In sexual behavior, this conflict could result in a failure to use condoms consistently, despite knowledge of the potential risks associated with unprotected sex. Adolescents who consume alcohol may be more likely to prioritize their immediate desires over their long-term health and wellbeing, leading to a failure to use condoms consistently during sexual activity. By understanding the role of inhibition conflict and alcohol expectancy in the association between alcohol consumption and inconsistent condom use, we can develop more targeted prevention and intervention strategies to reduce the gap of desires and conflict.

Although the rate of sexual experience among adolescents aged 13–18 years is increasing, only 65.5% of adolescents who have experienced sexual intercourse reported using condoms for contraception, with 64.6% being male and 67.0% female (11). This indicates that male adolescents are more passive in the practice of contraception compared to female adolescents. However, it is notable that alcohol consumption has a negative impact on condom use during sexual intercourse. The rate of condom use during sexual intercourse is already low at 57.9%, and drops to 51.2% when alcohol is involved (16). RSBs without condom use result in burden and responsibility due to unintended pregnancy among female adolescents. The integrative model of behavioral prediction (71) suggests that female adolescents may find it difficult to ask their partners to use condoms due to the influence of perceived normative pressure, lack of self-efficacy, and perceived control skill (14). To increase condom use among adolescents and prevent unwanted pregnancies or STDs after risky sexual intercourse, sex education should be provided in elementary schools regarding the consequences of not using condoms and how to use them correctly. Such education should be delivered not only within the school system but also at community centers and related institutions. Condoms should be universalized to enable youth to easily purchase them at pharmacies and convenience stores. Additionally, various programs should be implemented to strengthen communication, self-efficacy, and control skills so that female adolescents can confidently demand the use of condoms from their male partners.

The meta-analysis that examined the relationship between alcohol consumption and having multiple sexual partners included 25 studies and found that the risk of having two or more sexual partners was approximately 1.722 times higher for adolescents and young adults who drank alcohol. Among studies that examined the number of sexual partners by sex, Kugbey et al (34) and Mo et al (27) found that 61.1% of male adolescents had multiple sexual partners. However, some studies have reported no significant sex-related differences in the number of sexual partners (27). This study also found no significant sex-related differences depending on sex (male and female). The age at which alcohol consumption is initiated also affects the number of sexual partners. For example, a study found that females and males who began drinking alcohol at the age of 15 had a higher likelihood of having multiple sexual partners compared to those who began drinking at a later age (72). Such risky sexual behavior may expose adolescents to sexually transmitted diseases (STDs) through sexual contact with strangers. Therefore, it is important to educate adolescents and young adults on the dangers of having multiple sexual partners while drinking alcohol due to precocious puberty and open sexual culture. This could be accomplished through comprehensive sex education that covers the risks of unprotected sex, the importance of safe sex practices, and strategies for avoiding risky situations. It is also crucial to promote responsible drinking behaviors and discourage underage drinking, as early initiation of alcohol use is associated with a higher risk of engaging in risky sexual behavior. The Integrative Model of Behavioral Prediction (73) can be applied to understand the relationship between the intention to engage in RSBs and the actual practice of such behavior. This model may help in the development of a behavioral strategy to promote healthy sexual behavior. As alcohol negatively impacts all forms of RSBs, intervention for controlling adolescent drinking is necessary. However, since the level of risk associated with alcohol consumption varies depending on individual characteristics such as age and gender, it is difficult to apply the same guidelines for developing and implementing alcohol control programs from adolescents to young adults. Therefore, we propose developing intervention guidelines for each age group, considering factors such as age, gender, and educational methods, to develop the most effective guidelines. We also suggest using a tool called AUDIT (Alcohol Use Disorders Identification Test) to screen and assess risky drinkers among adolescents and young adults and provide different types of intervention based on their risk zone: alcohol education for low-risk drinkers, simple advice for hazardous drinkers, brief counselling for harmful drinkers, and referral for probable dependent drinkers (32).

### Limitations and Strengths

The study has several limitations, and we suggest some areas for future research. Firstly, the study did not take into account the frequency and amount of alcohol consumption, which varied among adolescents and young adults in each study. Additionally, the definition of variables such as the number of sexual partners and condom use varied across studies, with some studies measuring over a lifetime, and others measuring within a certain time frame (e.g., the last 60 days or the past 12 months). Therefore, we suggest that future meta-analyses should consider these variations in measurement when examining the relationship between alcohol consumption and risky sexual behaviors. Secondly, although the results indicate a positive association between alcohol consumption and RSBs in this population, it is notable that this association does not necessarily imply causality, as other factors may contribute to both behaviors. To address this limitation and better understand the causal relationship between alcohol consumption and RSBs, we recommend further research using longitudinal cohort studies or experimental designs. By following adolescents and young adults over time and controlling for potential confounding variables, these types of studies could help to establish the temporal sequence of alcohol consumption and RSBs and provide stronger evidence for causality. Finally, we only focused on three RSBs: early sexual initiation, inconsistent condom use, and multiple partners. However, there are other RSBs that can be influenced by alcohol consumption, such as casual sex, sex against the will, and sexual violence. Previous studies have suggested that alcohol can impair judgment and decision-making, increase sexual arousal and desire, and reduce inhibitions and self-control (74, 75). These effects can lead to engaging in sex with unfamiliar or untrusted partners, having unwanted or coerced sex, or being involved in sexual aggression or assault (76). Future research should examine how alcohol consumption affects these other RSBs among adolescents and young adults.

Despite these limitations, this study had several strengths. First, 20,000 articles were screened for studies on alcohol use and RSBs that reported odds ratios using two search strategies, and a large number of articles were comprehensively meta-analyzed. Three types of RSBs were analyzed to provide fundamental data for developing and implementing specific programs by comparing and discussing the risks of alcohol consumption among adolescents and young adults in early sexual initiation, which leads to inconsistent condom use and multiple sexual partners.

### Conclusion

This meta-analysis highlights the significant association between alcohol consumption and early sexual initiation, inconsistent condom use, and multiple sexual partners among adolescents and young adults. The findings suggest that alcohol consumption among adolescents and young adults may lead to exposure to unwanted pregnancy and STDs. Therefore, preventive interventions such as education and monitoring to prevent alcohol consumption and increasing the age criteria for drinking alcohol should be implemented. School- and community-based preventive interventions are required, such as education and monitoring to prevent alcohol consumption, and the age criteria for drinking alcohol in each country should be increased. Parents and guardians can provide guidance and support to help their children make healthy choices and avoid risky behaviors. Healthcare providers can also play an important role in preventing alcohol use among adolescents and young adults by screening for alcohol use, providing education on the harmful effects of alcohol, and referring adolescents and young adults to appropriate resources for prevention and treatment. Overall, it is crucial to raise awareness about the risks of alcohol consumption among adolescents and young adults and implement effective prevention measures to promote healthy behaviors and prevent the negative consequences of alcohol use.
